# Book Reviews

**Published:** 1812-04

**Authors:** 


*Practical Observations on various novel Modes of Operating on Cataract, and of forming an Artificial Pupil.* By Robert Muter, Holbeach, Member of the Royal College of Surgeons in London. 8vo. Wisbeach printed. 1811. pp. 115.

This is a treatise upon a subject, it must be allowed, of importance, but which has received so much elucidation as hardly to admit any novelty beyond that of method in treating the subject. But even this may be of great value. The terseness, perspicuity, and elegance, of one writer, may render clear, pleasing, and intelligible, what the confusion and dullness of another involves in darkness and obscurity.

The object of Mr. Muter is laudable. He intends "to produce an uniform mode of operating in cataract, and in forming an artificial pupil, to give as little disturbance as possible to the structure and natural situation of the parts of the eye:" and he aims at perspicuity by dividing his little book into thirteen sections.

The first of these treats on the opaque crystalline lens, its color, various consistence, and symptoms. The crystalline has been observed to become opaque first in its centre. Sometimes the opacity spreads rapidly, at others it is imperceptibly diffused: the diseased lens sometimes adheres to its capsule, and blood-vessels have been seen shooting upon it through that membrane. The color of the opaque lens is sometimes dark, at others pearly white. The most early and the most certain symptom of cataract, independent of the detectable opacity of the lens, is a settled mist, covering and confusing objects.

The second section is an enumeration of instruments; the third treats on "the mode of laying open the capsule of the lens, through a puncture in the cornea." The object of this operation is to admit the aqueous humor to act freely on the opaque crystalline. The steps of the operation are thus described.

"The patient is to be seated in a low chair, before a light not too bright and active; the eye not to be operated on covered by a compress retained by a bandage. The patient's head is then to be placed obliquely to a window, so that the eye to be operated on may be inclined towards the outer angle of the orbit. The operator, being conveniently seated, is to place the fore and middle fingers of the left hand upon the tunica conjunctiva, just below, and a little on the outside of, the cornea. At the same time the assistant, who supports the head, is to apply one, or, if the eye projects sufficiently, two of his fingers upon the conjunctiva a little on the inside and above the cornea. The fingers of the operator and assistant thus opposed to each other, will fix the eye and prevent the lids from closing closing. The eye being thus fixed, the point of the needle is to pierce the cornea in the line of its transverse diameter, immediately anterior to its connection with the sclerotica. It is then to be guided slowly forward, with its flat side parallel to the iris, until opposite the pupil. The point, being then turned backward, punctures the capsule of the lens near the border of the pupil on the same side it entered; and, passing a little way behind the capsule, not so far as to endanger the opposite side of the pupil being cut, its cutting edge is turned upward toward the capsule, and its point elevated. The cutting edge of the needle will divide that portion of the capsule between the point of the needle and where it first entered. Should it be thought proper to attempt to break down the lens, it would be more safe to withdraw this needle, and introduce one having a small curvature toward its point, and without a cutting edge."

The direct object of this operation, as just observed, is to admit the solvent properties of the aqueous humor to act upon the diseased crystalline, through an opening in its investing membrane, made in the manner above described. Professor Scarpa and Mr. Hey fully justify this operation, which with them has been generally successful. Sometimes the whole, at other times, if the crystalline has been broken down by the proper instrument, portions of that lens escape into the anterior chamber of the eye, but are soon dissolved and quickly absorbed. For some precautionary circumstances, and remedying infortunia as they may occur, we must refer to the work.

The extraction of a soft cataract, through a puncture or small incision of the cornea, is the subject of the fourth section.

The operation described in the preceding section, is considered, under particular circumstances, as preparatory to this of extraction. We have not room to transcribe Mr. Muter's description of the operation, but we must notice a point as we conceive of practical importance. It is that of preventing the escape, and leaving undisturbed the vitreous humor. This desirable object is effected by guarded and moderate pressure on the globe of the eye; and by carefully opening the anterior portion of the capsule of the lens only, leaving the posterior part in its natural state.

Section 5 is occupied by a "new mode of puncturing the capsule of the lens, and fixing the eye in the operation of extraction." The particular objects of this section are to show the advantages arising from puncturing the capsule of the crystalline, and admitting the aqueous humor to act, by its solvent powers, on the lens, sometime previous to extraction. By this, some difficulties are avoided in the operation, which occur when the section of the cornea is made previously previously to opening the capsule; and when the cataract is pulpy it may be dissolved and absorbed, rendering extraction unnecessary: and even a hard cataract may be diminished in size by a solution of some lamella of the lens. We see no other objection to this than what may arise from the patient's feelings. If he has been taught to believe that the whole will be performed at once, his disappointment will be great when he finds his eye is to be cut into again at another period. If the advantages gained are decided, this will not be a valid objection. The other part of the section describes an instrument by which the capsule may be punctured, the eye fixed without pressure on the ball, and the incision of the cornea made at the same time, with the method of using it. If, however, the author establishes his point, that the puncture in the capsule should be made some time previous to the incision of the cornea, we do not see why he should tax his ingenuity with the invention of an instrument that is to make the "puncture and incision of the cornea at the same time."

The sixth section describes "a new mode of extracting the cataract through an incision in the sclerotica."

"A puncture is to be made, by a couching needle, in the *sclerotica*, at the distance of little more than a line from its union with the cornea. Through this puncture a small hook is to be introduced, which is to be directed behind the capsule; so much only of it is to be introduced that its point, when brought forward, will reach the centre of the crystalline. If the length of the hook from its handle be little more than the diameter of the eye, the operation may be performed with great facility, being now very similar to couching. If, in bringing forward the point of the hook, the cataract is perceived to be pushed forward, and the pupil dilated, the operator will discover that it is of a firm consistence. When the hook has got sufficient hold of the cataract, that it may be removed from the axis of vision, the hook is to be withdrawn until resisted by the coats of the eye. The operator holding it, with the cataract affixed to its point, in the left hand, the patient's eye being turned inwards towards his nose, makes an incision with his right by a common scalpel through the sclerotica. The incision is to be begun at the puncture, and carried onwards, in a straight line, towards the external canthus. It should not be made directly into the eye, but in a slanting direction, through the coats. When the incision is of sufficient extent to permit the extraction of the cataract, it is to be withdrawn on the point of the hook. The lips of the wound should be neatly adjusted, and the eyelids closed."

The objection to this method is the formidable inflammation that will, probably, occur in the sclerotica. We are informed that our author is making experiments on inferior animals with a view to ascertain the degree of inflammation that may be expected to follow an incision in the sclerotica.

The extraction of the opaque lens, when adhering to the iris, is treated of in the seventh section. It frequently happens, that, after severe inflammation, not only the capsule but also the crystalline itself adheres to the iris. In such cases, though the incision of the cornea has been made of sufficient extent, and the capsule punctured, the lens does not escape from the eye. Nor will it be protruded through the pupil into the anterior chamber, even on the application of such pressure as might endanger the rupturing of the posterior portion of the capsule and membrane of the vitreous humour, or separation of the iris from the ciliary process. Our author recommends the removal of the adhesions by repeated attempts, rather than using much force at once. But we must refer to the section itself for a fuller explanation of his views.

A diseased condition of the vitreous humour is an impediment to the successful termination of operations for cataract. The infortunia attendant on this circumstance are the sudden sinking of the lens into the vitreous humor in attempts to lacerate its capsule, and, when a section of the cornea is made, the escape of that humor with the lens. The eighth section treats on this subject, and endeavors to guard against the above accidents.

The ninth section, which closes the first part of these "Observations," explains "the different modes of operating, by which the several kinds of membranous cataracts may be removed from the axis of vision, or extracted through a puncture in the cornea."

Three species or varieties of cataract are noticed in this section:—1st, The capsular, in which the opacity exists in the capsule of the crystalline. 2d, The lymphatic, formed by the coalescing of fragments, which remain after an operation. 3d, The reticulated, frequently a new production of a delicate fabric, resembling a spider's web.

The capsular cataract, or, as it is often denominated, with a reprehensible want of precision, the membranous, throughout the section, is described in many of its varieties, and the modes of operating laid down. The second and third species are barely noticed. We cite the following passage on account of its describing an ingenious, though perhaps too complicated, instrument.

"Sometimes, in secondary cataract, the vision is diminished by opaque fragments of the capsule, of a triangular shape, attached by their basis to the margin of the perforated capsule, and stretching their apices towards the centre of the pupil. When these fragments are are small, and the vision but little diminished, as considerable difficulty and danger of injuring the iris may attend any attempt to remove them, they should be suffered to remain. From the retraction of their apex, and being acted on by the aqueous humor, they generally in time become less. Whereas, when they are large, and, instead of being diminished, increase in size, they should be extracted as soon as this is perceived. These fragments may commonly be removed by puncturing their base with a small hook, and then gently turning it in different directions. But in some cases where the capsule adhered to the iris, they have been found to become thick and bulky, and forming a tough knob. In such cases they occasionally were not to be removed, even by the iris scissars. Did this occur so frequently as to merit attention, these knobs might be removed by an instrument resembling a flat canula, armed with a very fine silver or gold wire, and inclosing a little bistoury, having a cutting edge towards the end of the canula. This instrument being introduced through a puncture in the cornea, the wire is to be passed beyond the knob, and then drawn tight, so as to bind it to the end of the canula; the operator then slides the edge of the little bistoury beyond the end of the canula, and separates the knob from the capsule."

Connected with diseased crystalline, or consequent to an operation for removing that lens when opaque, and resulting from an inflamed eye, is an altered and deranged state of the iris, contracting and often closing the pupil. In this state of the iris, the operator sometimes succeeds in restoring vision by forming what has been technically termed an *artificial pupil.* It is the business of the remaining sections of this volume to point out the varieties of this altered structure of the iris, and to show how they may be removed by art.

Two general classes of causes give rise to defects of sight, which require the formation of an artificial pupil. One of these is confined to the iris, the other to the cornea. If the iris is completely closed by obliteration of the pupil, vision is lost; if it is contracted and partially closed, vision is diminished. If the cornea becomes impervious to the rays of light in the part opposite to the pupil, vision is impeded, diminished, or lost. In the tenth section, treating "on the different deranged states of the eye which may be relieved by an artificial pupil," the species or varieties of morbid alterations, arising from these causes, are explained.

"When the rays of light are prevented from falling on the retina, by the contraction or closure of the pupil, if the transparency of the cornea remain uninjured, a portion of the iris may be removed, and vision in a great measure restored. Total opacity of the cornea has hitherto been, and probably will for ever remain, irremediable; but, in these cases where a considerable part of it remains transparent, some some degree of vision may be restored by the removal of a portion of the iris, opposite that part of the cornea which retains its transparency."

On this principle the eleventh section proceeds to point out "the different modes of forming an artificial pupil."

Of all the operations performed upon the eye, those which are employed to remedy deranged and altered states of the iris, are the most difficult. The delicacy of structure, the mobility and the situation of the iris, concur to render precision and manual dexterity, in a superior degree, requisite in the operation. It is true, we believe, that our countryman Cheselden first ventured on this operation. Since that time a multitude of occulists have undertaken it, with shades of difference in their methods. Those particularly noticed by our author, are Janin, Maunoir, de Wenzel, Scarpa, and Gibson; to the latter of whom he gives the preference. The work of Mr. Gibson, on which this superiority is founded, was reviewed in one of our former numbers for the year 1811.

There are two conditions of the eye upon which this operation is founded. They respect the locality of the artificial opening: in one the opening is made in the natural situation, the centre; in the other, toward the margin, of the iris. The remaining sections 12 and 13, are appropriated to an explanation of these. For the detail we must refer our readers to the work.

Of the general merit of these "Observations," we must, every disadvantage considered, speak favorably. As issuing from the press of a small provincial town, we admit them to be indicatory of that spirit of literature which is pervading the most remote corners of the empire: and, though we cannot object to the investigation of any part or branch of the art, we are of opinion that by a close and critical examination of local circumstance, as they tend to preserve health or produce disease, as they modify morbid changes, or explain the operations of nature, more would be gained to science, than by laboring on subjects which require a greater space for observation than country practice presents.

---

*Edinburgh Medical and Surgical Journal, No.* XXIX.

*Observations on the Nature and Cause of certain Accidents which sometimes occur in Battle, and have been usually ascribed to the "Wind of a Ball."* By Daniel Ellis.—The subject of this paper is one of those obscure and mysterious matters that set men disputing, and leave them perplexed and in darkness. It might be admitted, *à priori*, that a large cannon ball, passing with immense velocity

 through through the atmosphere, might disturb, and possibly compress, portions of the aerial fluid to such a degree as to produce very sensible effects when opposed by, or impinging on, animal bodies: but, whether the effects, arising from this cause, are ever equal to the production of mischief to the extent many persons believe, we must at present express a doubt. By inserting some histories of this accident, what is meant by the injury arising from the "wind of a ball," will, we apprehend, be understood very clearly.

"There is, says Dr. Blane, (Observations on the Diseases of Seamen, 3rd. edit.) a singular species of accident, to which engagements at sea are liable, called, perhaps improperly, *the wind of a ball*. In whatever manner it is accounted for, it is a fact, that a part is sometimes severely hurt, and even life destroyed, without any visible external injury or breach of parts, or any appearance of the body from whence the injury proceeded. There were two instances, in the last battle,* of a ball passing close to the stomach, and producing instant death. The one was a lieutenant of the Royal Oak, the other a common sailor of the Bedford. A man in another ship, in consequence of a ball passing close to his belly, remained without sense or motion for some time, and a large livid tumor arose on the part, but he recovered. I attended a man at the hospital at Barbadoes, who had the buttons of his trowsers carried off by a cannon-ball, without any breach in the skin. The *pubes* was livid and swelled for some time after: he suffered exquisite pain from strangury, which seemed to proceed from a paralysis of the bladder, for he voided no urine without a catheter for nearly three months, after which time he recovered. I know a brave young officer† in the army, who had his epaulette carried off by a cannon-ball at Charlestown, in consequence of which, the shoulder and adjacent parts of the neck were affected for some time. A like accident happened to a marine officer in one of the late engagements; but in neither of these was the head materially affected, nor is it so apt to be affected in this way as the stomach. I never knew death the consequence of the wind of a ball on the head; though an officer‡ in the Sultan, at the battle of Grenada, was so stunned by a shot passing close to his temple, as to be insensible for some time, but he recovered entirely in a few hours."

"In some cases," continues Dr. Blane, "the bones sustained a severe injury from accidents of this kind. Two instances of it have come to my knowledge: the one was an officer, who fell down during an engagement without any obvious cause. Upon examination, the thigh was found to be broken, and the limb was two inches shorter, which seemed to proceed from the bone being pul-

* In the West Indies, April 1782.

† The Hon. Captain Fitzroy, now Lord Southampton.

‡ Colonel Markham.

verized, verized, as it were. There was no pain. The integuments were not in the least injured; so that this appears to have been what is called the "wind of a ball," but what ought, more properly, perhaps to be termed the *brush* of a ball. In the other instance, two of the false ribs were fractured and dislocated, with very little visible affection of the skin, though the clothes were torn. This accident proved fatal." Dr. Blane adds, that "animals are affected by these accidents as well as men. A cow in the Duke was killed in one of the actions in April, by a double headed shot passing close to the small of her back."

To these Mr. Ellis adds several other examples, in proof that serious and even fatal mischief does arise from a cannon-ball passing near a person, without absolute contact.

This effect from the "wind of a ball" has been denied, however, not by the uninformed only, but by persons of extensive knowledge and great acuteness. Mr. John Bell, noticing what he calls the prejudices of the present day, observes:

"It is, for example, believed, that even the whiff and wind of a ball, will extinguish life. I have heard sensible men of our profession affirm it. We find Belguer, the famous Prussian surgeon, perfectly convinced of it: and Tissot, in translating a book upon gun-shot wounds, sets gravely to prove, by many laboured calculations, how intense the force must be of that air which is pressed forward by a cannon-ball. This way of talking may suit very well an ignorant midshipman, or the coarse boatswain of a man of war; and many a good tale, no doubt, goes round in the cockpit about this wind of a ball: but it is unpleasant to observe men like Belguer talking so idly about this matter. Surely Belguer, of all people, might have known, that a man's right leg is often shot away, the breeches of the left thigh torn, and yet the thigh itself safe; and surely we must have seen the arm torn from a man's body, while his body has yet remained unhurt: how could a ball pass closer to the body, than in tearing off the arm? and when can this wind of a ball be dangerous if such a man escape? Surely Mr. Belguer must also have seen an officer's leg carried away by a shot, which had not hurt his horse; or a ball carrying off a man's arm, while his fellow who stood close up to him in the ranks received no hurt. Nay, still further, cases stand upon record, from the best authority, of soldiers whose arms have been carried away by the shoulder joint; yet they have suffered nothing but the loss of their arms, from which also they have recovered.

"But there is no report of this kind, however strange, which has not some meaning; and the reason of all these wonderful tales about the wind of a ball, is itself very wonderful. Men often fall in the field of battle, and when the camp followers come to turn over their bodies, in burying their dead, no wound nor mark of injury is seen; and often also men are laid in the military hospitals, dying dying and unable to speak, upon whom there is no kind of wound, nor even the slightest bruise of the skin.

"Let a ball hit any of the great cavities obliquely, and this phenomenon appears; the patient is killed without any external wound. He is killed, according to the notion of his fellow soldiers, by the wind of some great ball. But we know that the ball has actually struck him, that the breast, the belly, or the head, have been hurt. If the chest has been struck, then the ribs have perhaps yielded, and escaped the blow; but the lungs have suffered, and there is blood extravasated in the chest often, which suffocates the lungs; in the belly often there is a bursting of the liver or spleen, without any outward wound of the skin; very often in the head, though there appears no outward injury, the pericranium is separated from the scull, or there is an effusion of blood upon the brain."

With respect to the actual occurrence of this extraordinary and mysterious accident, thus then stands the evidence. Dr. Blane, and the author of this paper, admit or assert that it frequently does happen, even to the extent of producing death. Mr. John Bell considers it as an absurd prejudice, fit to act on only the ignorance of a boatswain, or a private soldier. We have, however, conversed with a physician of great professional skill, and who has been thirty years a surgeon in the navy; and with an army surgeon, now on the staff, who has been almost as long on actual service; both of whom speak of the accident as not uncommon, and that it is produced by some peculiar atmospheric commotion, resulting from the quick passage of a cannon-ball. The affirmative evidence certainly preponderates, and histories are so positively given as nearly to remove *our* scepticism.

Admitting then the existence of this phenomenon, we shall allow Mr. Ellis to state his opinion of its cause. It has hitherto, and generally we believe, been referred to a mechanical concussion, produced by the intense force of the air which is pressed forwards by a cannon-ball, moving with great velocity. But this is not sufficient to account for the effects produced, and Mr. Ellis looks to *atmospherical electricity* for a fuller explanation. He produces a number of instances where death has been occasioned by a stroke of lightning, and which seem very analogous to the effects produced by the wind of a ball.

"A man was suddenly attacked with failure of sight, and a sense of fullness and uneasiness in the stomach, which he first felt after being exposed to a flash of lightning: his sight gradually declined; and, although no alteration occurred in the state of his pulse or skin, he continued to grow worse, and died on the fifth day. On opening the body, all the viscera, except the stomach, were apparently rently sound; but that organ seemed to be in a mortified state. This state, it is added, was doubtless occasioned by the flash of lightning, and yet no mark was visible on the teguments of the body, through which the electric matter must have passed.* The loss of sense and motion, and the formation of livid marks, which have been ascribed to the 'wind of a ball,' are likewise common consequences of the operation of lightning. In one fatal case, recorded in the Philosophical Transactions, the electric matter entered the body under the left ear, and passed down the back, which it rendered as 'black as ink.'† In a second instance, sensation and circulation seemed to be suspended by a stroke of lightning, and it left a black mark on the thigh, which disappeared in a few days, and sensation also returned.‡ In a third case, a man was struck dead by lightning, and his body was rendered totally black: his clothes were torn, but no particular flesh-wound was made.§ The circumstances of the buttons being torn from the clothes, and the epaulettes carried away by the 'wind of a ball,' are likewise analogous to the effects sometimes produced by lightning; for, in the foregoing cases, the metallic substances, about the persons struck, was in some instances fused; in others it was torn from the clothes; and in others it remained uninjured. Lastly, the fracture of the bones, without injury to the skin, is also affected by the agency of lightning. Thus a person at Troyës was killed by lightning, and his bones were found broken, without any appearance of external injury. Muschenbröek relates a similar fact that came within his own knowledge. During a thunder-storm, the lightning struck a flock of sheep, which were all instantly killed, and their bones broken into minute pieces and dispersed through the flesh, without any external lesion.|| Extraordinary as these facts may seem, they will perhaps appear less wonderful to those who have experienced a smart shock from a common electrical machine, in which, although no effect is produced on the skin, a momentary sensation of fracture is communicated by the bones, arising, no doubt, from their imperfectly conducting power; while the soft parts, by yielding a ready transit to the subtile matter, impart no such feeling."

Upon the striking similarity of these facts to the effects from "the wind of a ball," the author founds his conclusion.

"The foregoing facts sufficiently prove," he says, "that all the peculiar effects, usually ascribed to the 'wind of a ball,' are likewise occasioned by the varied operation of atmospheric electricity; for, in both cases, persons are suddenly struck down with loss of sense and motion, vision is impaired or irreparably injured, the body is discolored, the nerves paralysed, the bones broken, and

* Simmons's Med. Facts, vol. viii.

† Phil. Trans. An. 1772, p. 135.

‡ Ib. 1773, p. 234.

§ Ib. 1781, p. 43.

|| Robertson's Hist. of the Atmosphere, vol. ii. p. 309.

even life destroyed, 'without any visible external injury or breach of parts, or any appearance of the body from whence the injury proceeded.' The fact, also, of metallic matter being torn from the clothes, without the ball having actually touched them, is a circumstance so truly electrical, as to be almost of itself sufficient to identify the nature of the agents by which such effects are produced. Indeed, it may be safely asserted, that many of the effects above mentioned, both in their nature and in the mode of their occurrence, are such as cannot be occasioned by any ordinary mechanical agent; and must therefore be attributed to the operation of electricity, or of some subtile matter, possessed of similar powers. Are there then sufficient reasons for believing that electric or other similar matter, exists in the air, and may be accumulated or developed, by the motion of a cannon-ball, in a quantity adequate to produce the extraordinary effects ascribed to the 'wind of a ball?'

"It is known that electric matter is abundantly diffused through the atmosphere, and, as we have seen, is often accumulated in a quantity sufficient to produce the most violent effects; but it cannot, I think, be reasonably maintained, that a cannon-ball, in its flight through so small a portion of air, is able, by friction or other means, to collect, from this source alone, electricity sufficient to produce the accidents ascribed to the 'wind of a ball.'

"Besides, however, yielding the electricity which may be diffused through it, many facts shew that a subtile matter, possessing electrical properties, may be developed from the air by the operations of chemical action. Chemists have amply proved, that all permanently elastic fluids owe their gaseous form to the presence of caloric in a latent state; and that, when the elasticity of the gas is reduced or overcome, its latent caloric is more or less set free. The oxygenous portion of our atmosphere contains a large quantity of this latent heat, and when, in combustion or other chemical operations, this gas loses its elasticity, its caloric is developed, and exhibited in a sensible state.

But this subtile or calorific matter, which oxygen gas is thus capable of affording, may be made to exhibit the properties of the electric fluid; for Dr. Wollaston found that this gas contributed to the production of electricity, by entering into combination with the amalgam of the rubber of the machine; and that, when the machine was duly insulated, no electricity was developed, either if the oxygen gas was withdrawn, or if an amalgam, incapable of oxydation, was employed:*—facts which seem clearly to prove, that the air contains, in a latent state, electric or other similar matter; and that it yields this matter in a sensible state, when, in these instances, it enters into *chemical combination.*

Farther, a subtile matter, resembling in its properties the electric fluid, is developed from the air, when its elasticity is reduced by *mechanical* means. By simply doubling the ordinary density of the air,

* Phil. Trans. 1801, p. 433.

air, Mr. Dalton found that caloric was disengaged sufficient to raise the temperature several degrees;* and in the greater and more rapid condensation to which the air is submitted in an air-gun, a flash of light is sometimes visible, as Mr. Fletcher observed.† M. Mollet farther remarked, that combustible substances were readily inflamed in a condensing syringe, by a few strokes of the piston;‡ and in subsequent experiments of M. Biot, oxygen and hydrogen gases, mingled in the proportions proper to form water, afforded, by their rapid condensation, subtile matter sufficient to inflame them, in the same manner, and with the same phenomena, as the electric spark operates.§ Without contending, therefore, for the absolute identity of caloric and the electric fluid, these facts must be allowed to prove that a subtile matter, exhibiting the appearances and possessing the distinguishing properties of that fluid, exists in gaseous bodies; and that it may be developed by reducing their elasticity, whether this be accomplished by chemical or mechanical means. Is it then probable that a cannon-ball, in its flight through the air, acts upon it in such a manner as to develop and accumulate a quantity of this subtile matter?

"By those philosophers who have particularly directed their attention to the motion of projectiles, we are informed, that elastic fluids, as that of the atmosphere, do not quickly fill up the space quitted by the body in motion, but condense more and more before it the quicker it moves. From the experiments of Dr. Hutton, it appears, that a cannon-ball, of about two inches in diameter, moving with the velocity of 1000 feet per second, encounters a resistance from the air equal to 350 ounces; while the resistance opposed to the same ball, when moving with a double velocity, is equal to 1569 ounces, or more than four times as great.‖ By this increasing resistance, and consequent condensation of the air, its latent caloric will necessarily be developed; and, as this subtile matter has been shewn, in so many of its properties, to resemble the electric fluid, it may, like it, be supposed to accumulate in the ball; and be carried forward with it, either till the velocity of the ball so much diminish as to permit its gradual diffusion, or till, like the electric fluid, it be suddenly drawn off, in the passage of the ball, by some less highly-charged conducting substance. In the latter event, it may, according to its intensity, produce those accidents already related; and hence the effects, usually ascribed to the wind of a ball, may be considered as, in their nature, truly electrical, and as really caused by the agency of the subtile matter developed by the condensation of the air, during the projectile's rapid motion.

"I may add, that those who have witnessed the effects of the ca-

* Manchester Memoirs, vol. v.

† Nicholson's Journal, vol. x. p. 280.

‡ Philosophical Magazine, xiv. p. 263.

§ Hauy's Traité de Phys. tom. ii. p. 253.

‖ Hutton's Phil. Dict. vol. ii. p. 365.

 lorific lorific matter, developed by the condensation of a few cubic inches of air, in a common syringe, will readily conceive what may be the operation of a 24 pounder, the section of whose great circle is about 25 square inches, and which, therefore, in a flight of 300 yards, or about *point-blanc* range, must, in half a second of time, act upon and displace 156 cubic feet of air.

"But this accumulation of subtile matter, which has been thus supposed to attend the rapid motion of a ball, can, in consistency with the principles above stated, occur only in the earlier period of its flight; since it then not only acts on a greater quantity of air in a shorter time, but this air, by its greater resistance then, also suffers greater condensation. At very small distances, also, it is probable that, although the *initial* velocity of the ball be so great, yet no considerable accumulation of subtile matter will take place, because sufficient space will not have been traversed to enable the air to afford it; while at very great distances, as the velocity declines, the resistance diminishes, and the air will then no longer be condensed in a degree sufficient to yield its latent calorific matter. It is possible also that, in a very moist atmosphere, no great accumulation will arise, because the conducting and dispersing property of the air, may then nearly or quite equal the condensing and accumulating power of the ball; while, in a very dry atmosphere, less subtile matter will be carried off, and more, therefore, will remain in the ball; and these considerations may, perhaps, explain the apparent fact of the more frequent occurrence of such accidents in the East and West Indies, than in Europe. Lastly, we may add, that most of the accidents ascribed to the 'wind of a ball,' seem to occur in sea-engagements, or in the operation of sieges, in both of which the contending parties fight often within *point-blanc* range, and the ball proceeds nearly in a horizontal course; but in land battles, from the greater distance at which the artillery is usually served, and the consequent elevation given to the piece, the ball is at once carried out of the horizontal path, and does not again come near its object, until its velocity is so much spent as to disable it from producing on the air those effects which attend its more rapid motion."

The analogy of effects, it must be allowed, and which are so remarkable in the instance before us, infer a similarity of cause: and perhaps electricity affords a rational explanation of a phenomenon hitherto involved in great obscurity.

---

*History of a Case of impracticable Labour, in which the Cæsarean Operation was performed; by* George Kellie, M.D. —In July 1811, a woman, at Leith, fell into labour, whose pelvis, at the longest diameter of its brim, measured, on the left side, one inch and $\frac{2}{10}$, on the right side $\frac{8}{10}$ of an inch only. Early in the morning, of the 3d of July, the labour commenced; about ten o'clock in the evening of the 4th, the Cæsarean operation was performed. The line of incision was on on the edge of the left rectus muscle, extending from two inches and a half above the navel, to four below it. The integuments being thus divided, to the extent of six inches and a half, the peritonæum, at the upper extremity of the incision, was opened by a probe bistoury to the same extent, as was the uterus;—the membranes were ruptured by the finger. The shoulders and neck of the child were exposed, and an elbow and arm immediately protruded; the presentation toward the pelvis was the breech. The child, a boy of full size, was born alive. The placenta was instantly and easily delivered; the uterus contracted vigorously; and no hæmorrhage occurred during the whole operation, either to embarrass the operator or affect the patient. There was a considerable protrusion of the intestines, which immediately followed the subsidence of the womb, and which were already affected with inflammation. The child died on the 5th, and the mother on the evening of the same day. We have thus one more fatal instance of this operation, added to the melancholy catalogue. It must never be forgotten, however, that this is an operation of necessity, and not of choice: and in this instance it afforded the only possible chance for saving the mother and child.

We subjoin the dissection and description of the pelvis:

"*Dissection.*—The abdomen appeared swollen and tense, but not discolored. The sides of the wound were in exact apposition, and externally no discharge or exudation could be perceived. Internally, there was a small circumscribed effusion of a coagulated lymph and blood between the peritonæum and subjacent bowels, immediately under and around the external incision. The intestines were vascular and pretty generally red and suffused from inflammation. There was a trifling effusion of bloody serum into the abdominal cavity. The uterus did not seem larger than what is natural at the same period after delivery. The sides of the incision were tumid, livid, and not in contact, but separated for half an inch. Its cavity contained no coagulum. The decidua was livid, easily rubbed off, and, with the lochia, extremely fetid. But the most interesting object of this dissection was to secure the pelvis, and to ascertain the nature and extent of the deformity which had opposed delivery by the natural passage, and authorised the section of the womb.

"*Description of the Pelvis.*—The last lumbar vertebra, and the upper portion or body of the sacrum, are protruded forwards and downwards into the cavity of the pelvis; and a part even of the fourth lumbar vertebra has fallen below the plane of the brim. The alæ of the ilia are folded or bent in such a way that each bone has the appearance of being formed of two planes, meeting at right angles: The inferior planes, or portions of the bones, with the acetabula, fall inwards upon the pelvis: And, immediately over the thyroidal foramina, the bodies of the ossa pubis are dissolved or fractured, and bent in such way that the iliac portions are also carried inwards with the ossa ilii upon the cavity of the pelvis, while the symphysis pubis is made to project outwards by the parallel approximation of the anterior halves of the bones. Thus, by the projection of the two lower lumbar vertebræ and sacrum, by the folding of the ossa ilii, and the fracture of the ossa pubis, the brim of the pelvis has become remarkably distorted, and lessened in all its dimensions.

"On the left side, the greatest linear distance between the ilium and spine is just one inch and two-tenths, stretching from above the acetabulum to the fourth part of the lumbar vertebra; and, on the right side, the space does not exceed eight-tenths of an inch. A line drawn across the fractured part of the ossa pubis, exactly over the thyroidal foramina, measures eight-tenths of an inch, and marks the approximation of the bodies of the two bones: and the distance between the middle of this line and the lumbar vertebra, which lies immediately behind it, is just one inch and two-tenths.

"The largest transverse diameters of this pelvis are lines extending from the fractures of the pubes to the opposite sacro-iliac symphyses; and are found to be on the right side four inches, and on the left four inches and two-tenths of an inch.

"The distortion of the outlet of the pelvis is no less remarkable than that of the brim, though its dimensions are not so confined.

"The rami of the pubes and ischia have been softened and thinned by absorption of osseous matter, and are much bent and distorted. The sacrum appears doubled upon itself, the lower half being suddenly inflected upwards, so that, with the coccyx, it fairly enters the pelvis, and becomes almost parallel to the superior part of the sacrum, at its junction with the inferior lumbar vertebra. The point of the coccyx is indeed exactly opposite to the cartilage which inter-articulates the sacrum and last vertebra of the loins; and the linear distance between the coccygial extremity, and this inter-articular cartilage to which it is opposed, is somewhat less than an inch.

"A line drawn from the point of the coccyx to the symphysis pubis measures exactly three inches and four-tenths: but, if carried no farther than the line stretching from the fracture of the pubes of one side to that of the other, and which line in truth completes the brim of the contracted pelvis, it does not exceed the length of two inches and two-tenths. From the spinous process of the ischium of the right side to the point of the coccyx, the distance is two inches: and from the spinous process of the left ischium, to the same point, just one inch. The space between the tuberosities of the ischia is two inches and seven-tenths; and between the spinous processes three inches and three-tenths.

"The bones in general, and more especially the alæ ilii, the ossa pubis, and the rami of the ischia, were rough and somewhat flexible; and the different pieces of which the pelvis is composed were loosely articulated, and to a certain extent moveable on each other."

---

*Case of Diabetes treated by Venesection; by* Mr. Murray, Surgeon,

Surgeon.—A man, aged 72, labouring under diabetes, lost fifty-four ounces of blood at four times in nine days, and was greatly relieved if not absolutely cured.

---

*Case of Metacarpal Bone successfully removed; by* J. Gregson, Surgeon.—This was removed by a method proposed, in 1808, by that excellent anatomist Mr. Brookes, and which, we believe, has since been adopted by the best surgeons.

---

*Case of Wounded Diaphragm. By* Alexander Boyle, A.M.—The conciseness of this history precludes abridgment.

"W. G., a man about 40 years of age, much addicted to the use of wine, was, on the 2nd of January, 1810, soon after dinner, suddenly seized with violent pain, which he referred to the epigastrium; and which was immediately followed by nausea and vomiting. I saw him, about an hour after he was attacked, when the inclination to vomit still continued, and he began to complain of cold and chilliness. The pain in the region of the stomach did not now appear so exquisite as at first; nor was it evidently increased by moderate pressure. There was no tension of the belly; but, on the contrary, I remarked an extraordinary hollowness, or drawing in, about the umbilicus. He complained greatly of thirst; his tongue was dry, and chopped, and had a bright glossy hue. He told me he had had a regular motion of his bowels the same morning. His skin was hot and dry; and the temperature about 98°. His pulse was full and hard, and 90 in the minute. Though the symptoms varied a little from those usually met with in gastric or intestinal inflammation, there still appeared such characteristic marks, as determined me, by a vigorous treatment, to guard against the fallacious nature which those complaints sometimes assume. Twenty-six ounces of blood were taken from the arm; and the evacuation would have been carried still farther, had I not been obliged to desist, on observing a tendency to deliquium coming on. He was put into a tepid bath; he took an ounce of *ol. ricini;* and a large enema was thrown up. The oil was immediately rejected; but, on his coming out of the bath, he had two copious stools. After this, the pain was not sensibly abated, and the smallest quantity of fluid, received into the stomach, was instantly rejected. In this state he continued the whole night. Towards morning, he had another evacuation by stool; he appeared cheerful, and said he felt no pain. These symptoms, however, did not deceive me; his pulse was scarcely to be felt, and cold drops of sweat bedewed his forehead, plainly announcing the fast approach of death, which happened soon after.

"*Appearances on Dissection.*—On opening the abdomen, there appeared an extraordinary derangement of parts. The stomach first presented itself, distended with fluid to a most enormous size, and occupying nearly the whole cavity. It appeared to be somewhat drawn drawn from its natural situation, and approached so closely to the pubes, that the fingers could scarcely pass between them. With the exception of a few minute red points externally, the stomach exhibiting no other marks of inflammation. The stomach being now raised up, the diaphragm presented a convex surface towards the abdomen. It was very tense; and the whole volume of intestines which is not bound closely to the spine by the mesentery, was found to have passed through a preternatural opening in the muscular part of the diaphragm, on the left side. On opening the thorax, it was found to contain several quarts of fluid, no doubt secreted on the vast surface of inflamed intestine, found lodged within that cavity; none, however, was found in the abdomen; its escape from the thorax being, no doubt, prevented by the pressure of the parts upon the orifice, acting in the manner of a valve. Both lobes of the lungs were of a pale color, and studded with small purple spots. The left lung was compressed into one-third of its natural size, and was confined to the upper part of the cavity, while the lower part of the chest was completely occupied by the displaced intestines. The duodenum shewed no marks of disease, and retained its natural situation. The jejunum, however, the whole of the ileum, (except about eight inches,) and the transverse portion of the colon, had passed through this preternatural opening. These parts were of a livid blue color, but of firm texture; and, on several parts of the mesentery and mesocolon, which were loaded with fat, there appeared large, red, and livid, blotches. The opening easily admitted two fingers; and the omentum, which had also passed through it, and adhered firmly to the adjacent parts, was convoluted round the colon in a singular manner, and nearly invested the whole of the protruded intestine, in a sort of pouch or sac. The edge of the orifice was smooth, and had a ligamentous hardness. It did not embrace the parts so firmly as to produce strangulation of the gut, and the intestinal tube was quite pervious.

"All around the margin of the opening, strong membranous productions of considerable length were observed, stretching out and forming attachments to the pleura costalis, the mediastinum, and some parts of the intestines. My attempts to account for the singularity of these appearances, brought to my recollection that this man, about eleven months before, had received a wound in the left side, between the sixth and seventh rib, passing obliquely downwards. He was brought to the hospital about midnight. A small portion of soft cellular membrane protruded from the wound. The wound was dilated in order to facilitate the return of the protruded part, but was healed in a few days, without the appearance of any bad symptom. About eleven months after he had received this injury, he complained of slight disorder in his bowels, but of which he soon recovered. This, I believe, was the first time he had complained since the wound was inflicted. From this time, however, he became less able to bear fatigue; he could not stand erect without feeling some uneasiness; respiration was affected; and even moderate exercise was supported with difficulty.

(To be continued.)

*A Treatise on some Practical Points relating to the Diseases of the Eye.* By the late J. C. SAUNDERS, Founder and Surgeon of the London Infirmary for curing Diseases of the Eye. *To which are added, a short Account of the Author's Life, and his Method of curing the Congenital Cataract.* By his Friend and Colleague, J. R. FARRE, M.D. The whole illustrated by colored Engravings, 8vo. pp. 216. Longman and Co. 1811.

(Continued from p. 236.)

*On the Congenital Cataract.*

CHILDREN are not unfrequently born blind, and the delicacy of the infant organ, with the unmanageable condition of infancy, have deterred occulists from an operation during the years of childhood. But, unfortunately, by delaying the operation, the value of it when performed has been incalculably diminished. Of the blessing of sight in such circumstances, it may be truly said, 'Bis dat, qui dat cito.' If we wait until the age at which reason may lend us her assistance, the vigour and beauty of the organ are lost. The eye as well as the mind requires a proper education. The retina, unused to the stimulus of light, gradually loses its sensibility—the motions of the iris, which determine the quantity of light proper for distinct vision—the mechanism, whatever it be, to which belongs the microscopic faculty—the adjustment of the organ to objects at different distances—the sympathetic motions necessary to perfect single vision—the entire control of the muscles, which enables us to fix or to move the eye as occasion requires—the habitual and unstudied expression of the passions, the 'mute eloquence which passeth speech'—all these are attained and confirmed by the early and healthy exercise of the organ.

It appears from the observations of Mr. Saunders, that the congenital cataract is commonly capsular, the substance of the lens being absorbed. This, however, is not the case where the opacity is partial; opacity is a condition necessary to absorption; and it appears that one form of the congenital cataract is that in which the opake centre is surrounded by a transparent margin. It is a melancholy fact that cataract is often congenital in two or more children of the same parents, and a curious one, that the texture of the cataract is similar in children of the same family. The Editor has given a table shewing the proportion of capsular to the other forms of the congenital cataract. Of forty-four cases, eighteen were purely capsular, three nearly so, some portion of lens remaining. The remainder solid, soft, or fluid, some with and some without opacity of the capsule, sule. The majority of these cases proving capsular, and the fact being ascertained by long observation, that where the lens remains, whether whole or in part, it undergoes solution in the aqueous humor, it became the object of Mr. Saunders "to effect a permanent aperture in the centre of the membrane."

"The excessive mobility of the eye, the unsteadiness of the little patient, the small field for the operation, and the flexibility of the opake capsula, are the difficulties with which the surgeon has to contend. The author overcame them by fixing the eye-ball with Pellier's Elevator, controlling the patient, dilating the pupil with the belladonna, and by using a diminutive needle, armed with a cutting edge from its shoulders to its point, and thin enough to penetrate with the most perfect facility."

The operation is either anterior or posterior to the iris, that is, the needle is passed through the cornea; or, as in couching, according to the more or less firm textuie of the cataract. The former is the least painful operation, and incurs the least risk of inflammation, the latter is that which gives the surgeon more power. If the cataract is centicular, the capsule must not be extensively rent, otherwise the lens is subject to dislocation, and this accident defeats in a great measure the design and advantage of the operation. Where the cataract is merely capsular, the surgeon may use a greater freedom with it. Children seldom suffer from subsequent inflammation.

The object of the operator, next to that of a central opening in the capsule, is to loosen the texture of the lens. The number of operations required, will depend on the texture of the capsule and the size of the lens. Frequently one, sometimes four, and rarely five, are required. On the same circumstances of course depends the period required for the cure, which varies from a few days to four or five months.

"The greatest success attended the operation between the ages of eighteen months and four years, and, if any intermediate time be selected, the editor is inclined to recommend the age of two years. The parts have then attained a degree of resistance which enables the surgeon to operate with greater precision than at an earlier period; yet the capsule has not become so tough and flexible as it does at a later period after the lens has been more completely absorbed."

The editor does not recommend the use of convex glasses, until the eye has gained by practice all that it can naturally acquire. "In the child," says Dr. Farre, "the preceding

 observations observations on the congenital cataract prove that Nature herself attempts the cure by the absorption of the lens: on this proceedure therefore, as the section of the cornea at so early an age is followed by the most unfavorable result, and depression cannot be accomplished, owing to the texture of the cataract, is founded the third operation; of which the essential part, as far as art is concerned, being the proper aperture in the capsule, it may be said to be *an operation on the capsule*, in contradistinction to extraction and depression, which imply principally the removal of the lens from its seat."

The operation in the capsule is the only one applicable to the condition of infancy; in the adult it is one of three. It may be substituted for couching or extracting the lens. Mr. Saunders first performed the posterior operation, using, in order to quicken the process, a larger needle than that which he ultimately employed; with this "he freely divided the capsule, and cut up the lens in its seat, disregarding its flocculi, or even small pieces which fell in abundance into the anterior chamber, even up to the margin of the pupil." The end was thus attained sooner, but frequently the inflammation was dangerous. On comparing this with the uniformly favorable result of the operation on the infant, the difference did not at first appear; but, upon more enlarged and strict inquiry, it resulted, that, "in the capsular cataract, inflammation very rarely followed the operation; in the lenticular, the inflammation was in proportion to the irritation or pressure which the iris sustained."

The operation, in its last state of improvement, was done with a considerably smaller needle, and confined to the centre of the capsule, with the view of freely exposing the lens to the action of the aqueous humor, while "a sufficient portion of the circumference of the anterior lamella of the capsule was preserved, to confine the lens in its seat."

In the firmest texture of the adult cataract, six or seven months are required for the process of solution. On this account the author appears to have been somewhat undecided between it and extraction in such cases. "It was intended," says the editor, "that such decision should result from a very long and impartial trial of both operations."

We are disposed to estimate highly the operation of Mr. Saunders. It is founded upon a series of well contrived experiments, the object of which was to restore vision with greater certainty. Accident, the prolific parent of discovery, presented him with a demonstration of his problem. A man punctured the healthy crystalline with an awl, it turned opaque, and he became blind; the aperture of the capsule was such as to expose the lens to the action of the aqueous fluid, the pupil cleared, and the patient saw. It seems that the progress of his experience led to restraint and reserve in his operation, and inculcated the wisdom of 'slow and sure.' He reduced the size of his needle: instead of tearing across the capsule, he made a hole in its centre; and, in place of slicing the lens to pieces, he only destroyed the compactness of its structure by the reiterated touches of his needle. This was the process of an observant and an ingenious mind; he labored to discern the imperfections of each method, that he might with certainty avoid them.

The concluding chapter, from which we have faintly sketched this outline of his practice, will be read with great interest. It presents us with an abundance of new and valuable matter, and is well illustrated by cases and plates. The whole of the work is written in chaste, simple, and forcible, language; and we must say, that, in the execution of it, the editor has discharged his duty to his friend and his profession in a manner that does equal honor to his character and talents.

We cannot conclude without briefly expressing our tribute of regard to the memory of Mr. Saunders. His mind was naturally fitted for sober observation, and had gained strength and acuteness by long and diligent exercise. He had received an education which qualified him for the highest department of the profession, and which enabled him to understand and explain appearances, and to reason upon what he saw. He was industrious, and so enthusiastically devoted to the pursuit which he had taken up, that it is to be feared his life was the sacrifice of his labors. Aided by the munificent citizens of the metropolis, he was the founder of one of its noblest charities, and the discoverer of a method, which he proved to be equally safe and certain, of restoring sight to persons born blind at the earliest period of infancy. If these things be true, where is the philanthropist who will refuse honor to his memory, where the man who will read the simple but affecting narrative of his life and death without emotion?

